# The NLRP3 Inflammasome Pathway: A Review of Mechanisms and Inhibitors for the Treatment of Inflammatory Diseases

**DOI:** 10.3389/fnagi.2022.879021

**Published:** 2022-06-10

**Authors:** Hallie M. Blevins, Yiming Xu, Savannah Biby, Shijun Zhang

**Affiliations:** Department of Medicinal Chemistry, Virginia Commonwealth University, Richmond, VA, United States

**Keywords:** NLRP3, inflammasome, NOD-like receptor, innate immune system, inhibitors, inflammatory diseases

## Abstract

The NLRP3 inflammasome is a multiprotein complex that plays a pivotal role in regulating the innate immune system and inflammatory signaling. Upon activation by PAMPs and DAMPs, NLRP3 oligomerizes and activates caspase-1 which initiates the processing and release of pro-inflammatory cytokines IL-1β and IL-18. NLRP3 is the most extensively studied inflammasome to date due to its array of activators and aberrant activation in several inflammatory diseases. Studies using small molecules and biologics targeting the NLRP3 inflammasome pathway have shown positive outcomes in treating various disease pathologies by blocking chronic inflammation. In this review, we discuss the recent advances in understanding the NLRP3 mechanism, its role in disease pathology, and provide a broad review of therapeutics discovered to target the NLRP3 pathway and their challenges.

## Introduction

Inflammation is a defense mechanism characterized by a cascade of signaling that activates the innate immune system in response to pathogens, dead cells, traumas, or chemically induced damage. The innate immune system involves immune cells that are derived from multipotent hematopoietic stem cells (i.e., hemocytoblasts) which are found in the peripheral blood and bone marrow and undergo hematopoiesis to give rise to myeloid and lymphoid progenitors (Kim S. et al., 2019). Myeloid progenitors differentiate into eosinophils, neutrophils, basophils, monocytes, and macrophages; whereas lymphoid progenitors give rise to cells of the lymphatic system such as natural killer (NK) cells, T-cells, and B-cells (Kim S. et al., 2019). While all immune cells play some role in inflammatory responses, the leading players are macrophages, neutrophils, NK, and T-cells during infections and injury. Immune cells, including neutrophils and monocytes, are recruited to the site of injury by chemotaxis (Kolaczkowska and Kubes, 2013; Chen L. et al., 2018). Neutrophils respond quickly and play important roles in the early stages of inflammation by eliminating pathogens through various mechanisms which subsequently leads them to become apoptotic and die. Once inflammation takes place, monocytes differentiate into macrophages and phagocytose the apoptotic neutrophils (Kolaczkowska and Kubes, 2013; Ortega-Gómez et al., 2013; Epelman et al., 2014). This process is tightly regulated, as an inadequate inflammatory response can result in a persistent infection of pathogens and an excessive inflammatory response may lead to chronic inflammation.

The recognition of pro-inflammatory stimuli by the germline-encoded pattern recognition receptors (PRRs) plays a pivotal role in initiating the innate immune response. PRRs can be classified as transmembrane or cytosolic receptors. Transmembrane proteins include Toll-like receptors (TLRs) and the C-type lectin receptors (CLRs) while cytosolic proteins include the nucleotide-binding oligomerization domain (NOD)- Leucine Rich Repeats (LRR)-containing receptors (NLRs), the Retinoic Acid-Inducible Gene 1 (RIG-1)-like receptors (RLRs), and Absence in Melanoma 2 (AIM2)-like receptors (ALRs) (Unterholzner et al., 2010; Lamkanfi and Dixit, 2014; Amarante-Mendes et al., 2018). These sensing receptors can recognize pathogen-associated molecular patterns (PAMPs) and damage-associated molecular patterns (DAMPs) (Chen and Nuñez, 2010). PAMPs represent the conserved structural moieties that are commonly found in microorganisms such as lipopolysaccharide (LPS) found on the membranes of Gram-negative bacteria, bacterial or viral nucleic acids, bacterial peptides such as flagellin, and polysaccharides such as β-glucans (Mahla et al., 2013). DAMPs are endogenous molecules which are released under cellular stress or damage such as chromatin-associated proteins, heat shock proteins, uric acid, and extracellular matrix fragments (Chen and Nuñez, 2010; Lamkanfi and Dixit, 2014). The activation of PRRs by PAMPs or DAMPs triggers a signaling cascade that causes cytosolic PRRs such as NLRs, ALRs, and tripartite motif-containing proteins such as pyrin to form the multimeric protein complex termed “the inflammasome” (Lamkanfi and Dixit, 2014). The inflammasomes play an intricate and vital role in the activation and release of pro-inflammatory cytokines. In the past two decades, the molecular components and mechanism of activation of different inflammasomes have been widely studied; however, the NLRP3 inflammasome is the most studied and characterized at present due to its ability to be activated by a diverse array of stimuli and its implication in several inflammatory diseases.

## NLRP3 Inflammasome Assembly and Activation

### Assembly

NLRP3 is a 118 kDa cytosolic PRR protein expressed by a variety of cells including neutrophils, macrophages, microglia, lymphocytes, epithelial cells, osteoblasts, neurons, and dendritic cells (Rada et al., 2014; Zahid et al., 2019). The NLRP3 protein contains a C-terminal leucine-rich repeat (LRR) domain, a central ATPase-containing NACHT (present in NAIP, CIITA, HET-E, and TP1) domain that mediates oligomerization, and an N-terminal pyrin (PYD) domain which recruits proteins for inflammasome complex formation (Kelley et al., 2019). Like other inflammasomes, the NLRP3 inflammasome complex consists of a sensor (NLRP3 protein), an adaptor (apoptosis-associated speck-like protein, ASC), and an effector (caspase-1) (de Zoete et al., 2014; Mamantopoulos et al., 2017). Formation of the NLRP3 inflammasome occurs in two stages: priming and activation. Priming is responsible for the transcriptional upregulation of NLRP3 and pro-inflammatory cytokines, pro-interleukin (IL)-1β and pro-IL-18, through TLR, NOD2, IL-1R, or tumor necrosis factor receptor (TNFR) ligand-mediated signaling upon stimulation with PAMPs and DAMPs such as LPS or cytokines such as tumor necrosis factor (TNF) and IL-1β. The detection of PAMPs and DAMPs results in the activation of proteins and nuclear factors such as myeloid differentiation primary response protein (MyD88), nuclear factor kappa-light-chain-enhancer of activated B cells (NF-κB), and the activator protein 1 (AP-1) to upregulate NLRP3 and pro-inflammatory cytokines (Liu et al., 2017). The effect of priming on transcriptional factors and protein expression has been widely accepted; however, recent studies have suggested that there is also a non-transcriptional role for priming (Lin et al., 2014). Priming also controls the post-translational modifications of NLRP3 such as ubiquitination and phosphorylation, which plays a major role in regulating the activation of NLRP3 (Yang et al., 2017). In its inactive form, ADP-bound NLRP3 can exist as a monomer or oligomer. Monomeric NLRP3 localizes to membranes which act as a scaffold to form an oligomeric, double-ring structure with 5–8 sets of dimers interlocking LRR domains and arranged back-to-back forming a circular cage (Andreeva et al., 2021; Hochheiser et al., 2021). Cryo-EM studies suggest the PYD domain sits inside the cage to protect it from aberrant activation (Andreeva et al., 2021).

A secondary stimulus initiates the activation step to form the active inflammasome complex. Contrary to most PRRs, NLRP3 is activated by a plethora of stimuli such as particulate matter (e.g., uric acid crystals, silica, asbestos), extracellular ATP, and toxins as well as viral, bacterial, fungal, and protozoan pathogens (Latz et al., 2013; Jo et al., 2016). Although it is unclear how NLRP3 can recognize such diverse signals, it’s suggested that NLRP3 senses a common cellular event caused by all stimuli rather than directly binding to them (Kelley et al., 2019). Chen and Chen (2018) found that disassembly of the* trans*-Golgi network (TGN) by multiple NLRP3 stimuli can recruit and activate NLRP3 and is now one of the proposed mechanisms for the detection of various diverse stimuli. Upon activation, NIMA-related kinase 7 (NEK7), an essential modulator of NLRP3 inflammasome activation and assembly, binds to NLRP3 (He et al., 2016). Recent studies suggest disassembly of the TGN causes the NLRP3 cage to localize on the dispersed TGN vesicle membranes which are then transported to the microtubule-organizing center where NEK7 resides (Magupalli et al., 2020; Andreeva et al., 2021). It is further hypothesized that the binding of NEK7 to NLRP3 disrupts the NLRP3 double-ring structure and causes structural rearrangement, exposing PYD domains, and allowing for NACHT domain oligomerization (Andreeva et al., 2021). Previous studies have placed the NEK7 binding site at NLRP3’s LRR domain; however, recent studies have shown that the LRR domain is dispensable for NLRP3 inflammasome activation and that NEK7 may have at least one additional binding site (Hafner-Bratkovič et al., 2018; He et al., 2018). Upon NACHT domain activation by ATP-exchange and NEK7 binding, the PYD domain then recruits the adaptor molecule ASC forming a filamentous complex referred to as ASC pyroptosome or “speck” clustering through PYD-PYD domain interactions. The caspase recruitment domain (CARD) of ASC binds to pro-caspase-1 which also contains a CARD domain (CARD-CARD interaction) and pro-caspase-1 is then converted into active caspase-1 by proximity-induced autoproteolysis (Lu et al., 2014; Lu and Wu, 2015; Malik and Kanneganti, 2017). Activated caspase-1 processes the biologically inactive peptides pro-IL-1β and pro-IL-18 into their active forms, IL-1β and IL-18, respectively. It also cleaves and activates the membrane pore-forming gasdermin D (GSDMD), a protein involved in the programmed cell death known as pyroptosis (Shi et al., 2015; Malik and Kanneganti, 2017). GSDMDs N-terminal domain (GSDMD-NT) shows high affinity to plasma membrane lipids cardiolipin and phosphoinositides. Upon cleavage, GSDMD-NT oligomerizes to form pores in the cell membrane. The subsequent disruption of the cell’s osmotic potential leads to pyroptosis and the release of intracellular contents, including inflammatory cytokines IL-1β and IL-18 (Ding et al., 2016; Evavold et al., 2018).

Activation of the NLRP3 inflammasome through canonical and non-canonical pathways has the same consequences, however, through different mechanisms. The non-canonical pathway results in inflammasome formation mediated by human caspase-4, caspase-5, and mouse caspase-11 (also called caspase-4/11) as a result of Gram-negative bacterial infection (Kayagaki et al., 2015). Extracellular LPS found on the membrane of Gram-negative bacteria is detected by TLR4 in conjunction with the adaptor protein TRIF (Rathinam et al., 2012). Similar to the canonical pathway, TLR4-MyD88 begins a downstream signaling cascade that results in the translocation of NF-κB into the nucleus to upregulate the expression of NLRP3, pro-IL-1β, pro-IL-18, and other inflammatory mediators (Liu et al., 2017). TRIF induces interferon regulatory factors (IRF) which then upregulates the expression of interferon (IFN)-α/β. IFN-α/β binds to IFN-α/β receptors which elicit caspase-4/11 expression through JAK/STAT signaling (Gurung et al., 2012; Rathinam et al., 2012). Another study reported that the protease Carboxypeptidase B1 is critical for caspase-11 expression and amplifies p38 MAPK signaling downstream of TLR4 and type I IFN signaling (Napier et al., 2016). Detection of extracellular LPS through TLR4 is not required for non-canonical inflammasome activation, however (Kayagaki et al., 2013). Type I IFN signaling also induces the expression of critical guanylate binding proteins (GBPs) which lyse intracellular bacteria and release LPS into the cytosol (Meunier et al., 2014). Intracellular LPS can be detected by caspase-4/11 which causes oligomerization and autoproteolytic cleavage to activate caspase-4/11 (Shi et al., 2014; Lee B. L. et al., 2018). Activated caspase-4/11 then facilitates pyroptosis through the cleavage of proteins such as GSDMD and regulating the release of IL-1α (Kayagaki et al., 2015; Shi et al., 2015; Wiggins et al., 2019); however, many of caspase-4/11 substrates have yet to be elucidated. Previous studies suggest caspase-4/11 may not have the ability to cleave pro-IL-1β and pro-IL-18 into their biologically active forms, unlike caspase-1, but still promotes NLRP3-induced caspase-1 activation and cytokine release through alternative pathways (Kayagaki et al., 2011; Py et al., 2014; Agnew et al., 2021).

### Activators

#### Ion Fluxes

Ion fluxes (K^+^, Ca^2+^, Cl^−^, and Na^+^) are suggested as one of the major mechanisms that trigger the activation of the NLRP3 inflammasome. Inflammasome activators such as extracellular ATP (*via* the non-selective cation channel receptor P2X7), particulate matter (aluminum hydroxide, silica, and calcium pyrophosphate crystals), and nigericin (ionophore) can induce K^+^ efflux, a necessary signal for NLRP3 activation (Muñoz-Planillo et al., 2013; Katsnelson et al., 2015). Monosodium urate (MSU) crystals cause cellular swelling from water influx resulting in an intracellular decrease of K^+^, leading to NLRP3 inflammasome activation (Schorn et al., 2011). Quinine, an inhibitor of two-pore domain K^+^ channels can prevent caspase-1 activity in a dose-dependent manner, highlighting the role of this family of ionic channels in inflammasome activation (Di et al., 2018). Conversely, K^+^ channel inhibitors Ba^2+^ (inhibitor of inward-rectifier K^+^ channels), tetraethylammonium (inhibitor of voltage-gated K^+^ channels), and iberiotoxin (inhibitor of large-conductance calcium-activated K^+^ channels) fail to prevent caspase-1 activation in macrophages primed with LPS (Di et al., 2018). Combined, these results leave questions and uncertainty about the role that potassium may have on inflammasome activation. The second messenger inositol 1,4,5-triphosphate (IP_3_) appeared to promote inflammasome activation by inducing efflux of Ca^2+^ from the endoplasmic reticulum (ER) through a ligand-gated ion channel, subsequently increasing intracellular concentrations (Murakami et al., 2012). The same study found that either depletion of ER Ca^2+^ by incubation with an inhibitor of sarcoplasmic/ER Ca^2+^-ATPase pump or incubation with Ca^2+^ free media to prevent extracellular Ca^2+^ entry attenuated NLRP3 inflammasome activation in response to ATP, suggesting mobilization of both extracellular and ER Ca^2+^ is required for inflammasome activation. Moreover, blockers of volume-activated Cl^−^ channels that are inactive against K^+^ channels are able to inhibit inflammasome activation, demonstrating the role of Cl^−^ efflux in NLRP3 inflammasome assembly (Compan et al., 2012; Daniels et al., 2016). It’s suggested that Na^+^ influx also plays a regulatory role in NLRP3 inflammasome activation in conjunction with K^+^ efflux induced by different stimuli. Na^+^ influx caused by ionophores cannot activate the NLRP3 inflammasome, however, lysosomal delivery of MSU crystals causes an increase in intracellular Na^+^ and water influx, subsequently lowering intracellular K^+^ concentration and activating the NLRP3 inflammasome (Muñoz-Planillo et al., 2013). More recently, the use of amiloride, a common epithelial Na^+^ channel blocker, can reduce cytokine secretion and caspase-1 activity in primary monocytes (Scambler et al., 2019).

#### Reactive Oxygen Species and Mitochondrial Dysfunction

Mitochondrial reactive oxygen species (mtROS) is one of the first discovered activators of the NLRP3 inflammasome and is produced during mitochondrial damage, stress, or dysfunction. Early studies suggested the role of mtROS in NLRP3 inflammasome activation when they noted the inhibition of NADPH oxidase-dependent mtROS prevents ATP-induced caspase-1 activation and IL-1β production in macrophages (Cruz et al., 2007). In addition, downregulation of voltage-dependent anion channels (VDAC), which are responsible for regulating mtROS homeostasis, results in decreased mtROS, caspase-1 activation, and IL-1β release in THP-1 cells (Zhou et al., 2011). Palmitate, a fatty acid commonly found in palm oil, is shown to lead to NLRP3 inflammasome activation in a ROS-dependent manner since the use of the ROS inhibitor APDC blocks palmitate-dependent IL-1β production (Wen et al., 2011). The complete mechanism by which NLRP3 senses mtROS is not well understood. Some studies suggest NEK7 may detect mtROS since it is an NLRP3 modulator (Shi et al., 2016). Supporting this idea, imiquimod, a TLR7 agonist, can activate the NLRP3 pathway and induce IL-1β release in a ROS-dependent manner, but fails to induce IL-1β production in cells deficient in NEK7 (Groß et al., 2016). Other studies have suggested mtROS’s involvement in the priming step, not activation, since some ROS inhibitors are able to block NLRP3 expression (Bauernfeind et al., 2011). Furthermore, mtROS is also implicated to be involved in the deubiquitination of NLRP3, subsequently promoting its activation (Juliana et al., 2012).

After the discovery of mtROS and its potential involvement in the inflammasome pathway, mitochondria in general have shown to play an intricate and important role in the activation of inflammasomes through various mechanisms. Mitophagy, the clearance of damaged mitochondria, is negatively correlated with NLRP3 activation, and deficiency causes the accumulation of dysfunctional mitochondria and the release of mtROS and mitochondrial DNA (mtDNA), both known activators of NLRP3 (Nakahira et al., 2011; Zhou et al., 2011). Further investigation revealed that other NLRP3 inflammasome activators, such as alum and nigericin, cause mitochondrial dysfunction and that oxidized mtDNA directly binds to NLRP3 (Shimada et al., 2012). Mitochondrial fission is also implicated in NLRP3 activation through LPS signaling; although the mechanism remains unclear, it’s suspected that fission facilitates NLRP3 localization and complex assembly (Park et al., 2015). The mitochondrial-specific lipid cardiolipin also plays a direct role in NLRP3 activation by binding to NLRP3 in a ROS-dependent and independent manner (Iyer et al., 2013). In addition, hexokinase, the first enzyme in glycolysis, dissociates from the mitochondrial membrane upon inhibition with bacterial peptidoglycans from Gram-positive bacterial cell walls and consequently activates NLRP3 (Wolf et al., 2016). Mitochondrial dysfunction and stress play a significant role in inflammation *in vitro* and *in vivo* (Gong et al., 2018; Mahalanobish et al., 2020; Ko et al., 2021); however, some studies dispute the requirement of mitochondrial dysfunction and the mtROS in NLRP3 activation (Muñoz-Planillo et al., 2013; Allam et al., 2014; Lawlor and Vince, 2014).

#### Lysosomal Damage

Lysosomes are responsible for degrading extracellular material by endocytosis or phagocytosis and degrading or recycling intracellular material via autophagy. Lysosomal damage is considered to play a significant role in NLRP3 activation, and direct evidence of this is shown when the lysosomal damage-inducing dipeptide, L-leucyl-L-leucine methyl ester (Leu-Leu-OMe), is able to activate the NLRP3 inflammasome (Hornung et al., 2008). In addition, NLRP3 activators such as particulate matter (e.g., cholesterol crystals, silica, aluminum salts, uric acid crystals, and asbestos) and misfolded proteins (e.g., amyloid-β) can induce lysosomal damage, indicating an interesting mechanism of NLRP3 activation (Emmerson et al., 1990; Martinon et al., 2006; Halle et al., 2008; Hornung et al., 2008; Duewell et al., 2010; Ito et al., 2020). There are several theories for how lysosomal damage activates NLRP3, one of the most popular being the release of cathepsin proteases, the proteins that mediate lysosomal degradation, into the cytosol where they can have apoptotic or apoptotic-like consequences (Wang F. et al., 2018) In support, genetic and pharmacological inhibition of cathepsin B impairs caspase-1 activation and IL-1β release in response to amyloid-β, Leu-Leu-OMe, and palmitate (Halle et al., 2008; Hornung et al., 2008; Weber and Schilling, 2014). However, no difference in caspase-1 cleavage or IL-1β secretion is observed with genetic or pharmacological inhibition of cathepsin B in response to MSU, hemozoin, alum, or ATP, suggesting a distinct mechanism for activators or cathepsin B (Halle et al., 2008; Dostert et al., 2009). In addition to this, cathepsins L, C, S, and X are also implicated to play a role in inflammasome activation (Orlowski et al., 2015). Other theories on how lysosomal damage induces NLRP3 activation include lysosomal acidification and influencing ion fluxes. Blocking the vacuolar H^+^-ATPase, a proton pump responsible for lysosomal acidification, prevents IL-1β release in response to silica, suggesting acidification is required (Hornung et al., 2008). In addition, lysosomal damage induced by Leu-Leu-OMe can affect K^+^ efflux and Ca^2+^ mobilization, subsequently activating the NLRP3 inflammasome (Murakami et al., 2012; Katsnelson et al., 2016). This also highlights the capability of NLRP3 activation pathways to work in conjunction.

## NLRP3 Inflammasome Dysregulation in Diseases

The pivotal defensive role of inflammasomes in response to pathogens and other danger signals also suggests inflammasome dysregulation in inflammation-mediated human diseases (Schroder and Tschopp, 2010; Liu and Chan, 2014; Moossavi et al., 2018; Boxberger et al., 2019; Eren and Özören, 2019). Aberrant NLRP3 activation has been shown to exacerbate disease pathology in several inflammation-driven diseases, and ongoing research is investigating the distinct role that NLRP3 may play in different disease states.

### Cryopyrin-Associated Periodic Syndromes

Cryopyrin-associated periodic syndromes (CAPS) are a group of diseases that are caused by a gain-of-function mutation(s) in the *nlrp3* gene and was the first autoinflammatory disorder to be directly linked to NLRP3 inflammasome dysregulation (Hoffman et al., 2001). Aberrant activation of NLRP3 causes recurrent episodes of fever, hive-like rashes, inflamed eyes, joint pain, swelling, headaches, and, if left untreated, deafness and amyloidosis (Kuemmerle-Deschner et al., 2017). CAPS include familial cold autoinflammatory syndrome (FCAS), Muckle–Wells syndrome (MWS), and neonatal onset multi-systemic inflammatory disease (NOMID) with symptoms and severity increasing respectively. Mortimer and colleagues found Ser295 in human NLRP3 is essential for negatively regulating NLRP3 activation via phosphorylation by protein kinase A, and mutations in adjacent residues interfere with regulation, rendering NLRP3 aberrantly active (Mortimer et al., 2016). A recent study showed that treatment of a knock-in mouse model expressing the N475K mutation of NLRP3 (in correspondence with the human N477K mutation) with the proton-pump inhibitor (PPI) esomeprazole is able to inhibit IL-1β secretion, reduce amyloid deposition, increase IL-1 receptor antagonist (IL-1Ra) production and survival rates (Bertoni et al., 2020). CAPS are incredibly rare genetic diseases and therefore have limited treatment options. Current treatment options consist of IL-1 inhibitors, however, the development of inhibitors that target the NLRP3 inflammasome is of great interest for these diseases.

### Diseases of the CNS

Alzheimer’s disease (AD) is characterized by the accumulation of amyloid-β (Aβ) and hyperphosphorylated tau tangles. Inflammation has been implicated to encourage the progression of AD, and activated inflammatory mediators and high levels of IL-1β are found in the serum, cerebrospinal fluid, and brain of patients with AD where it exerts neurotoxic effects against microglia and astrocytes (Rubio-Perez and Morillas-Ruiz, 2012; Parajuli et al., 2013; Saresella et al., 2016; Italiani et al., 2018; Ng et al., 2018). The accumulation of the Aβ peptide in lysosomes after phagocytosis by microglial cells leads to lysosomal swelling and destabilization causing the release of lysosomal contents, including cathepsin B, and activation of NLRP3 (Halle et al., 2008). The link between NLRP3 and AD pathology has been demonstrated in other studies as well, where genetic deficiency and pharmacological inhibition of NLRP3 in mice over-expressing human amyloid precursor protein (APP) and presenilin 1 (PS1) reduces Aβ deposition and improves cognitive functions (Heneka et al., 2013; Dempsey et al., 2017; Yin J. et al., 2018). Furthermore, microglia treated with Aβ causes ASC specks, a critical component of the NLRP3 inflammasome, but Aβ treatment failed to produce specks in cells with mutations in the PYD domain of ASC (Venegas et al., 2017). In addition, activation of NLRP3 causes hyperphosphorylation of tau in an IL-1β-dependent manner in Tau22 mice, suggesting NLRP3 works upstream (Ising et al., 2019). Clinical trials with drugs targeting Aβ and tau tangles have been unsuccessful, but NLRP3 has accumulated significant interest as a new target for AD.

NLRP3 inflammasome has also been suggested to play an important role in Parkinson’s disease (PD), a neurodegenerative disease characterized by the loss of dopaminergic neurons in the substantia nigra pars compacta, where high levels of IL-1β have been detected in patients suffering from PD (Mogi et al., 1994; Antony et al., 2013; Tan et al., 2020). The disturbed proteostasis and intraneuronal aggregation of fibrillar α-synuclein, known as Lewy bodies, interferes with neurotransmitter release and is a common hallmark of PD. These entities are able to activate the NLRP3 inflammasome through TLR2 and mitochondrial damage (Wang et al., 2020; Trudler et al., 2021). In addition, monocytes and microglia are able to clear α-synuclein which causes a robust release of pro-inflammatory cytokines, including IFN-γ, TNF, and IL-1β which results in neurodegeneration (Tan et al., 2020). Several studies have supported the link between NLRP3 and PD pathology. NLRP3 deficient mice are resistant to the loss of dopaminergic neurons in the substantia nigra after treatment with 1-methyl-4-phenyl-1,2,3,6-tetrahydropyridine (MPTP), a neurotoxin that induces a PD-like phenotype (Yan et al., 2015; Lee E. et al., 2019). In conjunction with this, pharmacological inhibition of NLRP3 improved PD pathology (Gordon et al., 2018). Prolonged exposure of IL-1Ra knockout mice to cytokines IL-1β/IL-1α results in PD-like outcomes, including impaired motor skills (Stojakovic et al., 2017). Together, this highlights the role of NLRP3 and cytokines in PD.

Multiple sclerosis (MS) is an autoimmune neurodegenerative disorder caused by the demyelination of neurons presumably by reactive T-cells that infiltrate the CNS upon weakening of the blood-brain barrier (BBB). NLRP3 and IL-1β have long been implicated to play a role in MS through encouraging immune cell infiltration and promoting excessive inflammation. Caspase-1 and IL-1β are found in MS plaques and are upregulated in peripheral blood mononuclear cells of MS patients (Ming et al., 2002; Cao et al., 2015). In experimental autoimmune encephalomyelitis (EAE), an animal model for MS, NLRP3 inflammasome expressing antigen-presenting cells assist in T-cell migration to the CNS through upregulation of chemotaxis-related proteins (Inoue et al., 2012). In addition, NLRP3 expression is upregulated in the spinal cords of mice with EAE, and NLRP3 and ASC knockout mice are resistant to EAE, highlighting their potential roles in MS (Gris et al., 2010; Inoue et al., 2012). IFNβ, one of the few therapeutic options for MS, can suppress NLRP3 activation and in NLRP3-dependent EAE (Inoue et al., 2016).

Neuroinflammation is the standard response to traumatic brain injury (TBI), and several studies have supported the upregulation of inflammatory mediators, including NLRP3, in the brain hours to days after a TBI (Liu et al., 2013; Wallisch et al., 2017). Cytokines IL-1β and IL-1α’s RNA are elevated as soon as 3 h after TBI in rats, and inhibiting the two cytokines with antibodies prior to TBI significantly reduces the loss of hippocampal neurons (Lu et al., 2005). NLRP3 and other inflammatory mediators are also upregulated in the brain of patients after suffering severe TBI, and recent studies reveal pharmacological inhibition of NLRP3 decreases cell death and prevents neurological deficits in TBI mouse models, highlighting the druggable potential of NLRP3 (Chen et al., 2019; Kuwar et al., 2019; Yan et al., 2020). TBI’s link to AD and PD is well established, and other studies support that patients with a history of TBI are at increased risk for neurodegenerative diseases in general (Gardner and Yaffe, 2015; Hayes et al., 2017; Ramos-Cejudo et al., 2018; Delic et al., 2020). These increased risks are likely contributed to neuroinflammation, as neuroinflammation after TBI shows increased hyperphosphorylation of tau protein and Aβ plaques, two hallmarks of AD (Johnson et al., 2010; Edwards et al., 2020).

### Peripheral Inflammatory Diseases

Dysregulation of NLRP3 inflammasome has also been suggested in the pathogenesis of rheumatoid arthritis (RA), a chronic autoimmune disease characterized by persistent synovial inflammation in small diarthrodial joints, progressive cartilage, bone destruction, and autoantibody production (McInnes and Schett, 2011). Numerous studies support the involvement of NLRP3 in the development of RA, however, the mechanism is somewhat elusive. One study suggests pentaxin 3, a biomarker of RA, and its ligand C1q can activate NLRP3 (Wu et al., 2020). Significant NLRP3 expression is observed in the synovial proliferation and subchondral vasculitis areas in the paws of collagen-induced arthritis (CIA) mice compared to healthy mice (Zhang et al., 2016). Similarly, pharmacological inhibition of the NLRP3 inflammasome with different inhibitors reduces RA pathology and secretion of IL-1β and TNF-α, supporting the involvement of NLRP3 in RA (Yan et al., 2012; Voon et al., 2017; Guo et al., 2018; Marchetti et al., 2018b).

Gout has many of the same symptoms as RA, however, the pathology of these two diseases is different. While RA is an autoimmune disease, gout is caused by elevated levels of uric acid in the bloodstream. Eventually, these uric acid crystals accumulate in the joints where they are phagocytosed by synoviocytes (Richette and Bardin, 2010). Upon phagocytosis, uric acid crystals cause lysosomal destabilization which subsequently activates NLRP3, cytokine release and causes inflammation and pain (Martinon et al., 2006). In addition, soluble uric acid was able to activate NLRP3 in a ROS-dependent manner, suggesting inflammation is initiated before crystal formation (Braga et al., 2017). Several NLRP3 inhibitors have been shown to inhibit inflammation caused by gouty arthritis, emphasizing the importance of NLRP3 as a target in treatments for gout (Lee H. G. et al., 2016; Ruiz-Miyazawa et al., 2017; Huang et al., 2018; Marchetti et al., 2018b; Lee H. E. et al., 2019; Deng et al., 2020).

Diabetes mellitus is another disease in which NLRP3 dysregulation has been suggested to play a pathological role (Chausmer, 1998; Tang et al., 2019). High levels of IL-1β have been detected in patients with diabetes and other studies show IL-1β can affect insulin sensitivity through the TNF pathway (Dinarello et al., 2010; Wen et al., 2011). Transgenic mice lacking NLRP3 fed with a high-fat diet, which has previously been shown to activate NLRP3, have lower IL-1β and are protected from high-fat diet-induced insulin resistance, further supporting inflammatory mediators’ involvement in metabolic inflammation and insulin resistance (Vandanmagsar et al., 2011; Wen et al., 2011). Recently, it was reported that NLRP3 inflammasome inhibition could be one of the mechanisms underlying metformin’s effects to inhibit diabetes-accelerated atherosclerosis (Tang et al., 2019). Consistent with this observation, the blockade of IL-1 receptor using anakinra, a drug used for RA, leads to significant improvements in diabetic patients (Larsen et al., 2009).

At least 20% of all cancers arise from infection or chronic inflammation (Grivennikov and Karin, 2011). Chronic inflammation is led by the release of pro-inflammatory cytokines; however, while NLRP3 is an important mediator in inflammation, reports show that NLRP3 can have both a destructive and protective role in different types of cancer, making NLRP3’s role in cancer complex (Hamarsheh and Zeiser, 2020). Several studies have shown that NLRP3 and components of the NLRP3 inflammasome pathway can play a destructive role through upregulation, overactivation, and polymorphisms (Hamarsheh and Zeiser, 2020). One example of this is IL-1 and TNF’s ability to recruit neutrophils, encouraging ROS production and subsequent inflammation as well as inducing adhesion molecules and metalloproteases which encourage tumor invasion (Dinarello, 2006). Polymorphisms and mutations in the NLRP3 gene are also implicated in cancer. Poor survival rate is correlated with invasive colorectal cancer patients that have the *Q705K* NLRP3 mutation, a mutation that is also prominent in pancreatic cancer patients (Ungerbäck et al., 2012; Miskiewicz et al., 2015). Conversely, NLRP3 is also demonstrated to have anti-tumorigenic effects in colorectal cancer. One study found that mice deficient in NLRP3 or caspase-1 exhibit high sensitivity to azoxymethane (AOM) dextran sodium sulfate (DSS)-induced inflammation and suffer tumor burdens as a result of decreased IL-18 and lack of tumor suppressing cytokines, IFN-γ and single transducer and activator of transcription-1 (STAT-1) (Zaki et al., 2010b). Along those same lines, inflammasome component deficiency also correlates with a worse disease state in colitis-associated cancer (CAC) as a result of decreased IL-18 (Zaki et al., 2010a). The exact role of NLRP3 in different types of cancer is multiplex, but therapies that target inflammation may be of value to some diagnoses.

### NLRP3 Inflammasome Pathway Inhibitors

**Figure 1:**
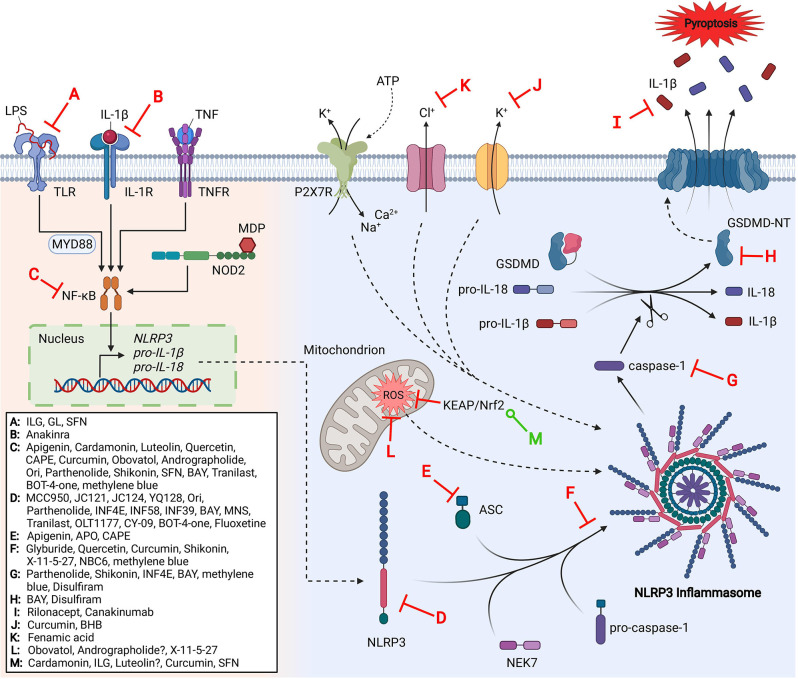
The NLRP3 inflammasome pathway and its inhibitors.

The link between NLRP3 inflammasome dysregulation and a variety of human diseases has suggested the value of NLRP3 inhibitors for therapeutic interventions. Several NLRP3 inflammasome inhibitors have recently emerged and been investigated in a variety of disease states; some of which have entered clinical trials. Herein, we summarize the mechanisms of inhibition and relevant SAR studies that have been conducted for direct and indirect inhibitors of the NLRP3 inflammasome (Figure 1).

### Sulfonylurea and Sulfonamide Analogs

**Figure 2:**
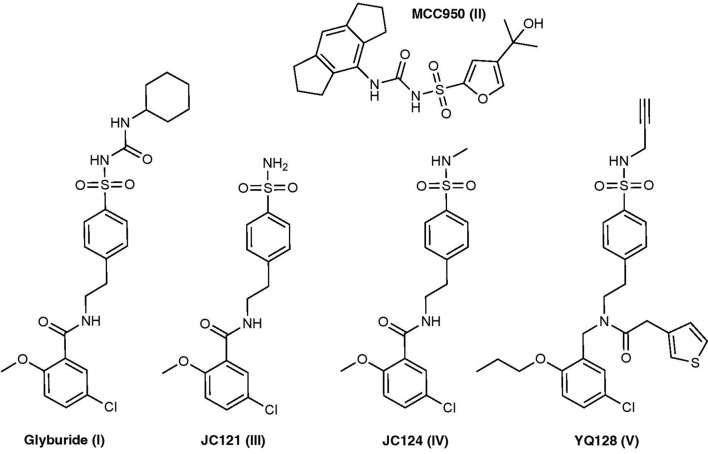
Sulfonamide-based NLRP3 inhibitors structures.

Inflammasome inhibitors containing the sulfonylurea and sulfonamide moieties have been developed and studied to improve potency, selectivity, and solubility (Figure 2).

#### Glyburide^®^

Glyburide is an FDA-approved, anti-diabetic drug that falls into the sulfonylurea class of medications. Glyburide treats diabetes by blocking ATP sensitive potassium (K_ATP_) channels in pancreatic β cells (Ashcroft, 2005). Studies have shown that glyburide inhibits NLRP3 activation and IL-1β secretion induced by LPS/ATP in bone marrow-derived macrophages (BMDMs) with an IC_50_ of 10–20 μM (Lamkanfi et al., 2009; Hill et al., 2017). Lamkanfi et al. (2009) demonstrated that glyburide inhibits caspase-1 activation and IL-1β secretion independently of K_ATP_ channels and later concluded that glyburide likely targets downstream of the purinergic 2X7 receptor (P2X7R) and upstream of inflammasome formation. Pilot structure-function studies revealed that the cyclohexylurea group is responsible for insulin secretion, whereas the benzamide and sulfonyl moiety are critical for NLRP3 inflammasome inhibition (Hill et al., 2017). Unfortunately, the concentration that glyburide is able to elicit its anti-inflammatory properties can induce hypoglycemia which limits its further development as an NLRP3 inflammasome inhibitor (Coll et al., 2015).

#### MCC950

MCC950 is a sulfonylurea compound initially discovered in 2001 under the name CP-424, 174 (Perregaux et al., 2001). The term CRID3 is also used synonymously with MCC950. Recent studies have demonstrated that MCC950 is a potent and selective NLRP3 inflammasome inhibitor that can inhibit IL-1β release in BMDM cells with an IC_50_ of 7.5 nM (Coll et al., 2015; O’Neill et al., 2016). However, it should be noted that in a 2011 article by Coll et al. (2011), a different compound was incorrectly termed CRID3 and was described to be nonselective and inhibiting both NLRP3 and AIM2 inflammasomes. Mechanistic studies revealed that MCC950 is a direct NLRP3 inhibitor that binds to the Walker B motif of the ATPase binding pocket in the central NACHT domain subsequently inhibiting ATPase activity, a critical step in inflammasome activation (Coll et al., 2019). Further docking studies supported this binding site (Tapia-Abellan et al., 2019; Andreeva et al., 2021; Hochheiser et al., 2021). MCC950 fails to stimulate insulin secretion *in vitro*; however, a later study found it’s able to increase insulin sensitivity in an *in vivo* diabetic mouse model, although this was attributed to MCC950’s effects at NLRP3 (Hill et al., 2017; Zhai et al., 2018). In 2017, Hill et al. (2017) synthesized a series of MCC950, sulfonylurea derivatives that can inhibit NLRP3 inflammasome with nanomolar potencies and also inhibit insulin secretion. Since the discovery of MCC950 as an NLRP3 inhibitor, it’s been employed as a chemical tool to understand the pathological roles of the NLRP3 inflammasome in a variety of disease models including AD, atherosclerosis, asthma, allergic airway inflammation, inflammatory bowel disease (IBD), and many more (Dempsey et al., 2017; van der Heijden et al., 2017; van Hout et al., 2017; Ismael et al., 2018; Perera et al., 2018; Robinson et al., 2018; Xu et al., 2018; Zhai et al., 2018; Theofani et al., 2019; Fu et al., 2020; Ren et al., 2020). The commonly used dose in these studies was 10 mg/kg, relatively high compared to its low nanomolar potency. Recent studies of radio-labeled MCC950 PET tracer demonstrate its low CNS penetration, which may limit its development and use as a CNS agent (Hill et al., 2020).

#### JC121, JC124, and YQ128

Formerly known as 16673-34-0, JC121 was developed as a sulfonamide derivative of glyburide. JC121 is able to inhibit ASC aggregation, caspase-1 activity, IL-1β secretion, and inflammatory cell death in cardiomyocytes in response to ATP and nigericin (Marchetti et al., 2014). Furthermore, JC121 inhibits the activity of constitutively active NLRP3 in BMDMs from genetically modified mice, suggesting that this compound inhibits downstream inflammasome activation rather than upstream targets (Marchetti et al., 2015). Notably, JC121 does not show hypoglycemia effects *in vivo*, a concern for its scaffold predecessor glyburide, even at doses as high as 500 mg/kg. This compound exhibits *in vivo* protective activity in mouse acute myocardial infarction models and preserves cardiac function after ischemic injury (Marchetti et al., 2014, 2015). To overcome the observed solubility issues of JC121, another analog, JC124, was designed that incorporates a methylated sulfonamide into the structure. The results demonstrated that, like JC121, JC124 is also a selective inhibitor of the NLRP3 inflammasome that inhibits IL-1β release with an IC_50_ of 3.25 μM without significant inhibition on NLRC4 or AIM2 inflammasomes (Fulp et al., 2018). Mechanistic studies employing a photo-affinity probe suggested that JC124 directly interacts with the NLRP3 protein; however, unlike MCC950, JC124 can bind to NLRP3 without affecting the ATPase activity, suggesting a distinct mode of binding for these sulfonamide derivatives (Kuwar et al., 2019). *In vivo*, JC124 can actively reduce disease pathology and improve functional performance in animal models of TBI, AD, and acute myocardial infarction by engaging the NLRP3 inflammasome (Fulp et al., 2018; Yin et al., 2018; Kuwar et al., 2019). Structure-activity relationship (SAR) studies of JC124 revealed that the sulfonamide moiety can be modified to improve inhibitory potency.

To increase the space for structural optimization, a new chemical scaffold was created based on the structure of JC124 and further medicinal chemistry campaign led to the design of YQ-II-128 (YQ128) as a novel NLRP3 inhibitor (Jiang et al., 2019). YQ128 significantly inhibits NLRP3 mediated IL-1β production *in vitro* with an IC_50_ of 0.30 μM in mouse macrophages. Further *in vitro* studies demonstrated YQ128 as a selective inhibitor of the NLRP3 inflammasome, since it did not inhibit IL-1β production mediated by NLRC4 or AIM2 inflammasomes. *In vivo* studies also confirmed its selective inhibition on IL-1β production upon LPS challenge in mice. A YQ128 PET tracer showed rapid BBB penetrance with moderate brain uptake; however, preliminary pharmacokinetic studies revealed that YQ128 displays poor oral bioavailability (Jiang et al., 2019; Xu et al., 2021). Ongoing SAR studies are currently underway to improve the potency and pharmacokinetics of these compounds.

### Natural Products

**Figure 3:**
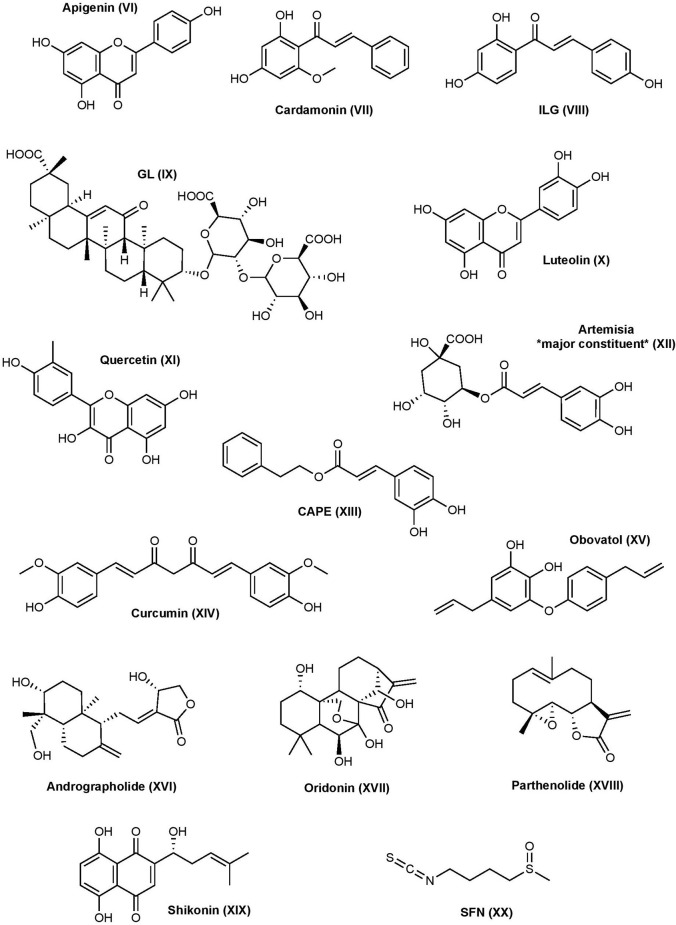
Natural product NLRP3 inhibitors structures.

Numerous natural products possess anti-inflammatory activity with mechanisms and targets that go far beyond the breadth of this review. However, there are several natural products that have received considerable attention for their anti-inflammatory activity being attributed to the interference of inflammasomes and their pathways (Figure 3).

#### Flavonoids

Flavonoids are a group of phytonutrients found in a variety of fruits, vegetables, grains, and plants and are well recognized for their neuroprotective, anti-inflammatory, antimutagenic, and antioxidant properties.

##### Apigenin

Apigenin is a flavone that inhibits NLRP3-mediated inflammation through a variety of different mechanisms (Zhang et al., 2014). The expression of pro-inflammatory cytokines IL-6, IL-1β, and TNF-α are reduced by apigenin in LPS-primed THP-1 derived macrophages and J774A.1 mouse macrophages. Apigenin shows inhibitory activity on the ERK1/2 and NF-κB pathways. Despite apigenin inhibiting NF-κB activation, it does not affect NLRP3 mRNA or protein levels or ASC protein levels in response to LPS; however, apigenin inhibits ASC speck formation and further studies suggested this happens through specifically targeting ASC (Zhang et al., 2014). A recent study demonstrated that apigenin can inhibit the phosphorylation of spleen tyrosine kinase (Syk) and protein tyrosine kinase 2 (Pyk2), two tyrosine kinases that are key players in the phosphorylation of ASC (Lim et al., 2018). The same study found apigenin can inhibit AIM2-mediated IL-1β production, but not NLRC4-mediated, in THP-1 cells, making apigenin a non-selective inflammasome inhibitor. Apigenin presents therapeutic potential in several disease states, but more research is needed (Salehi et al., 2019).

##### Cardamonin

Cardamonin is a natural chalcone found in the plant *Alpinia katsumadai* Hayata. Cardamonin can inhibit the NF-κB pathway through suppression of nitric oxide (NO) and prostaglandin-E_2_ in IFN-γ- and LPS-induced RAW 264.7 cells (Israf et al., 2007). This results in decreased phosphorylation and degradation of Iκ-Bα and subsequent activation of NF-κB. Other potential mechanisms of cardamonin include upregulating AhR/Nrf2/NQO1 signaling, which is known to negatively regulate NLRP3 (Wang K. et al., 2018). So far, cardamonin has mainly been investigated in cancer, but it has also been studied in IBD, RA, and more (Voon et al., 2017; Wang K. et al., 2018; Jin et al., 2019; Liao et al., 2019).

##### Isoliquiritigenin and Glycyrrhizin

Isoliquiritigenin (ILG) and Glycyrrhizin (GL) are both extracts from the plant *Glycyrrhiza uralensis* (licorice) which have been used throughout time to treat a variety of disorders mainly due to their abundance in flavonoids, chalcones, and other phytonutrients. ILG is a flavonoid that resembles a chalcone, while GL is a triterpene saponin. Both ILG and GL inhibit the NF-κB and MAPK activation by suppressing TLR4/MD-2 complex (Honda et al., 2012). ILG has also been recognized to promote the Nrf2 pathway and suppress ROS (Zeng et al., 2017). Further studies show that GL and ILG inhibit NLRP3 inflammasome formation, activation of caspase-1, and production of IL-1β when added during the activation step (Honda et al., 2014). Collectively, the results suggest that ILG and GL can inhibit both the priming and activation steps of the inflammasome pathway. Furthermore, ILG is a selective inhibitor of the NLRP3 inflammasome, as it did not inhibit AIM2 inflammasome formation or IL-1β production in response to poly (dA:dT). Contrarily, GL shows inhibitory activity to both NLRP3 and AIM2 inflammasome mediated IL-1β production (Honda et al., 2014). Because ILG has selectivity for NLRP3, and ASC oligomerization is a shared mechanism between NLRP3 and AIM2, ILG may target NLRP3 directly or somewhere upstream to inhibit inflammation. Because GL is not selective, GL may target ASC or somewhere upstream.

##### Luteolin

Luteolin is a flavone that can be identified in plants including pepper, broccoli, thyme, and celery (Imran et al., 2019). Mechanistically, luteolin can reduce ROS and expression of NLRP3 and other inflammatory mediators through the NF-κB pathway; however, it is a nonspecific inflammasome modulator as it also affects the expression of AIM2 (Chen et al., 2007; Zhang et al., 2018; Yu et al., 2019). One of these studies showed that luteolin potently inhibits NLRP3 expression as low as 2 μM in RAW267.4 cells and attributed this effect to its ability to enhance M2 macrophage polarization which is known to stimulate immunoregulation (Zhang et al., 2018). Further research suggested the potential involvement of transcription factor Nrf2 in the observed inhibition of inflammasome activation (Hennig et al., 2018). Luteolin and some derivatives have been considered as a treatment for several diseases including ulcerative colitis (inflammatory bowel disease), several types of cancer, and myocardial injury (Ning et al., 2017; Imran et al., 2019; Li B. et al., 2021).

##### Quercetin

Quercetin is a flavonol that is found in many fruits, vegetables, leaves, grains, and more (Bentz, 2009). Quercetin is a non-selective inflammasome inhibitor that inhibits IL-1β secretion during NLRP3 and AIM2 challenging (Domiciano et al., 2017). During the priming step, quercetin can inhibit NF-κB and MAPK as well as JAK/STAT pathways under AIM2 challenging (Hämäläinen et al., 2007; Lee K. M. et al., 2018). Other studies have indicated that quercetin inhibits the activation step of the NLRP3 inflammasome by interfering with ASC-speck oligomerization, which may explain why it cannot inhibit NLRC4 since ASC is dispensable for NLRC4 activation (Domiciano et al., 2017). Quercetin has been investigated in neurodegenerative disorders, cancer, diabetes, and more (Chen et al., 2016; Amanzadeh et al., 2019; Reyes-Farias and Carrasco-Pozo, 2019; Zaplatic et al., 2019).

#### Phenols

##### Artemisia

The genus *Artemisia* contains a variety of species, some of which have been used in traditional Asian medicines for their anti-inflammatory properties. Extract of *Artemisia princeps* (APO) contains several phenolic compounds such as caffeoylquinic acids, chlorogenic acid, and neochlorogenic acid; however, the anti-inflammatory properties are mainly attributed to chlorogenic acid which is one of the major constituents in the APO extract. APO can inhibit ASC speck formation upon activation of the NLRP3 or AIM2 inflammasomes, but not NLRC4, in BMDM cells (Kwak et al., 2018). The same study demonstrated that APO interferes with the phosphorylation of a tyrosine residue (Y144) of ASC, which is critical for speck formation. This is consistent with its dual inhibition on the NLRP3 and AIM2 inflammasomes since ASC is not required for the assembly and activation of the NLRC4 inflammasome (Kwak et al., 2018). *Artemisia* and their extracts have become an attractive area of natural product medicine and several other species have been investigated for their anti-inflammatory properties and suspected roles in the inflammasome pathways (Chen et al., 2021; Manayi et al., 2021; Wang Q. et al., 2021).

##### Caffeic Acid Phenethyl Ester

Caffeic acid phenethyl ester (CAPE) is a phenolic compound found in propolis from honeybee hives. Similar to the other phenolic compounds, CAPE can suppress the activation of transcription factors, specifically NF-κB, that leads to a decrease in the production of pro-inflammatory mediators (Natarajan et al., 1996; Juman et al., 2012). CAPE has recently been shown to inhibit the activation of NLRP3 and AIM2 inflammasomes by binding to ASC and blocking NLRP3-ASC interactions (Lee H. E. et al., 2016). It was later revealed that CAPE binds to the ASC-PYD domain, but not to the ASC-CARD or NLRP3-PYD domain. Because NLRP3 and AIM2 both require ASC, CAPE likely has a similar mechanism toward AIM2. CAPE has been investigated in gout, cancer, Lou Gehrig’s disease, neuroprotection in status epilepticus, and diabetes (Fontanilla et al., 2012; Yis et al., 2013; Fraser et al., 2016; Lee H. E. et al., 2016; Nie J. et al., 2017).

##### Curcumin

Curcumin is a widely used polyphenol compound known for its anti-inflammatory and antioxidant effects. A 2016 study found that treatment with curcumin decreases the expression of NLRP3, activation of caspase-1, and cleavage and secretion of IL-1β in PMA-induced macrophages (Kong et al., 2016). The reduced expression of NLRP3 is likely due to its inhibition of IKK phosphorylation, which is required for NF-κB activation; however, the expression of several other pro-inflammatory mediators was also reduced such as TLR4 and MyD88, suggesting multiple targets which is consistent with the promiscuous nature of this compound. Curcumin is also able to reverse P2X7R activation in PMA-induced macrophages, subsequently decreasing the expression of NLRP3 (Kong et al., 2016). Later studies found that curcumin is selective to the NLRP3 pathway, as it does not inhibit NLRC4- or AIM2-mediated caspase-1 activation in LPS-primed BMDMs (Yin H. et al., 2018). Other than priming, curcumin also suppresses ASC speck formation, caspase-1 activation, and IL-1β secretion in LPS-primed BMDMs, suggesting it affects both the priming and activation steps. This study also found that curcumin can inhibit potassium efflux in macrophages and inflammasome NLRP3 complex formation. Despite ROS being one of the activators of NLRP3, curcumin’s antioxidant effects are not the primary mechanism responsible for its inhibitory effect on the NLRP3 inflammasome pathway, however, it is recognized to upregulate the Nrf2 pathway (Yin H. et al., 2018; Rahban et al., 2020). Curcumin itself has been investigated in a variety of different diseases including, but not limited to, AD, osteoarthritis, depression and anxiety, cancer, and many more (Lopresti and Drummond, 2017; Sun et al., 2017; Reddy et al., 2018; Giordano and Tommonaro, 2019).

##### Obovatol

Obovatol is phenol isolated from the bark of *Magnolia obovata*. Like other natural biphenolic compounds, obovatol is traditionally used for its anti-inflammatory, anxiolytic, and nootropic characteristics. Obovatol can inhibit NF-κB, JNK, and ERK pathways, resulting in the suppression of inflammatory mediators (Ock et al., 2010). In addition, obovatol can also block the production of mitochondrial ROS and the formation of ASC inflammasome in response to nigericin and dsDNA, suggesting it inhibits both NLRP3 and AIM2 inflammasome activation (Kim J. et al., 2019). Further studies are required to understand the mechanism of action and binding. Obovatol has been investigated for use in AD and cancer (Choi et al., 2012; Duan et al., 2018).

#### Terpenes

##### Andrographolide

Andrographolide is a diterpenoid extracted from *Andrographis paniculate* and is commonly prescribed as a treatment for RA, asthma, laryngitis, and other upper respiratory tract infections (Tan et al., 2017). It is known to suppress the NF-κB and MAPK pathways and may also act as a ROS scavenger in ovalbumin-induced lung injury (Peng et al., 2016; Nie X. et al., 2017). Andrographolide can also inhibit NLRP3 activation by stimulating mitophagy, a process that is negatively correlated with the inflammasome, in colitis-associated cancer (Guo et al., 2014). Recently, andrographolide has been shown to also inhibit the AIM2 inflammasome by preventing AIM2 from translocating into the nucleus to sense DNA damage during the development of radiation fibrosis in BMDM cells (Gao et al., 2019). Andrographolide has been investigated in several diseases, however, due to its effects on AIM2 it is been closely investigated for its antiviral properties (Gupta et al., 2017; Reshi and Chi-Yong, 2020; Shi et al., 2020).

##### Oridonin

Oridonin (Ori) is a natural diterpenoid from the plant *Rabdosia rubescens* that is commonly used in East Asia as a natural supplement for its anti-inflammatory, anti-tumor, antimicrobial, and neuroprotective effects (Xu et al., 2018). Studies have shown that Ori can suppress the level of several pro-inflammatory mediators by inhibiting NF-κB signaling, nuclear translocation, and DNA binding (Wang et al., 2014; Cummins et al., 2018). Contrarily, increased expression of pro-TNF-α and increased phosphorylation of IκB in L929 cells have also been observed by Ori treatment, which may contribute to pro-inflammatory effects (Huang et al., 2005). Recent studies showed that Ori is a direct, irreversible, and selective inhibitor of NLRP3 and inhibits IL-1β production with an IC_50_ of ~0.5 μM (He et al., 2018). Mechanistically, Ori is able to disrupt NLRP3-NEK7 interactions, a crucial interaction for NLRP3 activation and inflammasome assembly, by covalently modifying the cysteine residue C279 in the NACHT domain of NLRP3 protein by Michael addition. Further studies showed that Ori failed to inhibit NEK7-NEK9 interactions, suggesting Ori inhibits the NLRP3-NEK7 interaction by binding to NLRP3. Ori shows no effect on the ATPase activity of NLRP3, NLRP3-NLRP3 interactions, or recruitment of ASC (He et al., 2018). Ori has demonstrated *in vitro* and *in vivo* success and has been investigated in a number of disease models including TBI, AD, cardiac hypertrophy, methicillin-resistant Staphylococcus aureus (MRSA), pulmonary fibrosis, cancer, and more (Wang et al., 2016; Fu et al., 2018; Xu et al., 2019; Yuan et al., 2019; Zhang et al., 2019; Yan et al., 2020). Despite the observed *in vivo* activities of Ori from preclinical studies, there is concern about the toxicity and adverse pharmacological effects of Ori (Li X. et al., 2021). More research on Ori analogs is required to improve pharmacological activity and bioavailability.

##### Parthenolide

Parthenolide is a natural product found in *Tanacetum parthenium* and has commonly been used as a preventative for migraine headaches. Its anti-inflammatory activity has been attributed to its ability to inhibit the NF-κB pathway, however, it also shows anti-inflammatory effects independent of the NF-κB mechanism (Saadane et al., 2007). One study has demonstrated that parthenolide inhibits multiple inflammasome pathways including NLRP3, NLRP1, and NLRC4 and directly inhibits caspase-1, but does not inhibit the AIM2 pathway (Juliana et al., 2010; Coll et al., 2011). Conversely, one study demonstrated that parthenolide does not inhibit NLRC4 (Coll et al., 2015). Mass spectrometry results show that parthenolide irreversibly inhibits caspase-1 by alkylating C285 of the p20 subunit, blocking activity and cleavage of downstream events. It is suspected that parthenolide block NLRP3 in a similar manner due to its ability to inhibit ATPase activity in a dose-dependent manner (Juliana et al., 2010). Parthenolide can inhibit IL-1β secretion with an IC_50_ of ~5 μM in response to NLRP3 challenging and 5–10 μM in response to AIM2 and NLRC4 challenging in BMDM cells (Jiang H. et al., 2017). Naturally occurring parthenolide’s poor aqueous solubility and bioavailability limits its further clinical development, but due to its reactive species it is still being investigated in different disease, states including, cancer and analogs continue to be made in an effort to improve pharmacological properties (Guzman et al., 2007; Alwaseem et al., 2018; Li et al., 2020; Liu et al., 2020; Hotta et al., 2021).

#### Other

##### Shikonin

Shikonin is a naphthoquinone compound isolated from the roots of *Lithospermum erythrorhizon* and is used in traditional Chinese herbal medicine for its variety of medicinal properties. Shikonin inhibits the secretion and cleavage of pro-caspase-1 through suppression of NF-κB as well as directly inhibits caspase-1 itself, likely by targeting a cysteine residue in the active site (Zorman et al., 2016). Furthermore, shikonin was able to inhibit NLRP3-mediated IL-1β secretion with an IC_50_ of 1.4–2 μM in LPS primed iBMDMs. Another study demonstrated that shikonin prevents ASC pyroptosome formation and NLRP3- and AIM2-mediated IL-1β and IL-18 release in BMDM and THP-1 cells through inhibition of PKM2, a kinase that promotes the activation of NLRP3 and AIM2 inflammasomes (Xie et al., 2016). Shikonin has been investigated in the treatment of AIDS and cancer (Chen et al., 2003; Wang et al., 2017, 2019; Zhao et al., 2018).

##### Sulforaphane

Sulforaphane (SFN) is a widely used dietary supplement that is found in broccoli extracts and cruciferous vegetables. SFN is known to have anti-inflammatory properties that are attributed to activating the Nrf2 transcription factor, a protein that regulates the antioxidant responses upon oxidative damage caused by ROS, and NF-κB by employing its ability to modify cysteine residues (Heiss et al., 2001; Jiao et al., 2017). SFN can also inhibit TLR activation through covalent modification of a cysteine residue in the hydrophobic pocket of myeloid differentiation 2 (MD2), which complexes with TLR (Koo et al., 2013). However, recent research has shown that SFN has anti-inflammatory properties independent of the priming step and is a non-selective inflammasome inhibitor of the NLRP3, NAIP5/NLRC4, NLRP1b, and AIM2 pathways (Greaney et al., 2016). SFN can inhibit NLRP3-mediated IL-1β production with an IC_50_ of 5 μM and NLRC4- and AIM2-mediated IL-1β production with an IC_50_ of ~10 μM (Jiang H. et al., 2017). It’s not clear whether SFN acts in a direct or indirect manner; however, the non-selective inhibition on multiple inflammasomes may suggest that SFN targets a shared mechanism by the inflammasomes during the activation process. Greaney et al. (2016) also showed that the SFN inhibits caspase-1 autoproteolytic cleavage but does not modify cysteine residues in the caspase-1 active site, as incubation with SFN in the presence of active caspase-1 does not inhibit the IL-1β processing. SFN itself has been investigated for the treatment of retinal ischemic/reperfusion injury, cancer, neurodegenerative diseases, and many more diseases (Kan et al., 2018; Gong et al., 2019; Kim, 2021). There is abundant research on the dietary benefits of broccoli in preventing diseases, and SFN is suggested to play a significant role (Subedi et al., 2019; Nandini et al., 2020; Liebman and Le, 2021).

### Other Inhibitors

**Figure 4:**
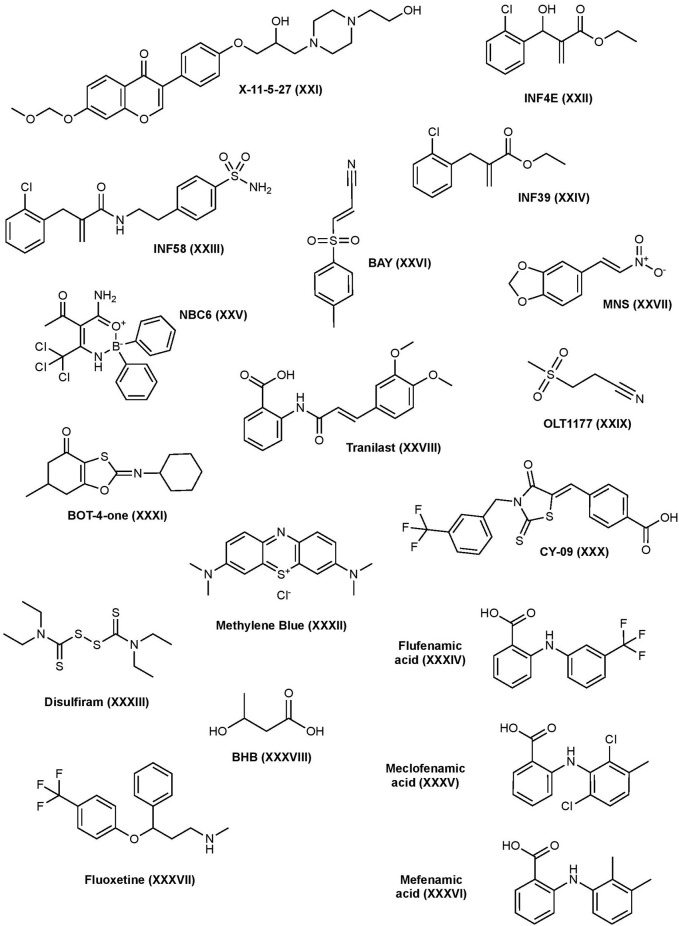
Other NLRP3 inhibitors structures.

Several synthetic small molecules and biologics have been investigated to inhibit the NLRP3 inflammasome pathway as well, some of which have successfully made it to the market (Figure 4).

#### X-11-5-27

X-11-5-27 is a synthetic derivative of daidzein, a natural isoflavonoid found in soybeans, with antioxidant and anti-inflammatory properties. Daidzein has been shown to suppress the secretion of pro-inflammatory cytokines *in vitro* and it’s suspected that these effects are the result of a variety of targets in the MAPK pathway (Hämäläinen et al., 2007; Liu et al., 2009; Sakamoto et al., 2016). X-11-5-27 inhibits NLRP3 expression and activation, ASC speck formation, and subsequent caspase-1 activation and IL-1β secretion in BMDMs and THP-1 cells (Zhou et al., 2017). It was also observed that X-11-5-27 inhibits NLRP3 through the induction of autophagy, a process that is negatively correlated with NLRP3. Further studies suggested that the inhibitory activity of X-11-5-27 in these studies may be through antioxidant effects by reducing ROS production and protecting mitochondrial function (Zhou et al., 2017).

#### INF Analogs

In 2014, a series of α, β-unsaturated warheads from an electrophilic fragment-based library were reported to irreversibly inhibit the NLRP3 inflammasome (Cocco et al., 2014). Further characterization of one of the compounds, INF4E (compound 9), demonstrated its irreversible inhibition of the NLRP3 protein via its Michael acceptor to form a covalent bond with NLRP3, which subsequently inhibits IL-1β secretion in ATP-induced THP-1 cells. INF4E also inhibits NLRP3’s ATPase and caspase-1 activity, suggesting those as targets. Furthermore, INF4E prevents caspase-1 dependent pyroptosis and attenuates ATP- and nigericin-induced cell death (Cocco et al., 2014). To decrease toxicity associated with these types of compounds, further structural derivatization led to a series of acrylamide analogs using the ligand-merging strategy to combine the Michael acceptor moiety and a sulfonamide moiety. One of the analogs, INF58 (compound 14), irreversibly inhibits NLRP3 ATPase with improved potency (Cocco et al., 2016). Further optimization led to the identification of an ethyl acrylate derivative, INF39 (compound 11), that possesses decreased cytotoxicity and reactivity compared to both INF4E and INF58. Mechanistic studies suggest its direct interaction with the NLRP3 ATPase domain and can inhibit IL-1β production with an IC_50_ of 10 μM (Cocco et al., 2017). The same study found that INF39 can attenuate NEK7-NLRP3 interactions in HEK293 cells expressing NLRP3 and NEK7, confirming that INF39 blocks NLRP3 inflammasome assembly. INF39 continues to be investigated in the inflammasome pathway, a recent study has further verified INF39’s selective interaction with NLRP3, as it does not suppress AIM2 or NLRC4 inflammasomes, and showed the inhibition of ASC speck formation and the cleavage of caspase-1, IL-1β, and GSDMD proteins in THP-1 cells (Shi et al., 2021). Other studies have continued to derivatize INF39 and investigate the mechanism of binding (Gastaldi et al., 2021).

#### Novel Boron Compounds (NBC)

2-aminoethyldiphenyl borate (2APB), a boron containing compound, is known to inhibit IP_3_-mediated calcium release, store-operated calcium entry, and disrupt other calcium-dependent pathways in HeLa cells and cardiac myocytes, which may affect the ion flux balance required to activate NLRP3 (Peppiatt et al., 2003). Further studies demonstrated that 2APB can inhibit nigericin- and ATP-induced NLRP3 inflammasome signaling cascade independently of its effect on calcium flux (Katsnelson et al., 2015). However, 2APB’s effects on calcium flux limits its drug development. Further optimization of 2APB to weaken its effects at calcium flux while strengthening the effects on NLRP3 resulted in compound NBC6 with significantly enhanced potency (IC_50_ = 574 nM) (Baldwin et al., 2017). NBC6 can inhibit ASC oligomerization and was found to be a potent inhibitor of NLRP3; however, it’s also able to inhibit NLRC4 and AIM2 pathways at higher concentrations. Interestingly and unlike 2APB, NBC6 retained activity after washing away free drug, suggesting it may act as a covalent inhibitor. NBC13, a similar analog to NBC6 with better solubility, inhibited IL-1β release *in vivo* (Baldwin et al., 2017). NBC compounds have yet to be investigated in disease states.

#### BAY 11-7082

BAY 11-7082 (BAY) is a phenyl vinyl sulfone and is known for its inhibition of TNF-α induced IKK phosphorylation which subsequently inhibits activation of NF-κB and expression of NLRP3 (García et al., 2005). However, due to its non-specific modification of cysteine residues, BAY shows polypharmacological properties with multiple mechanisms suggested (Lee et al., 2012). In 2010, a study found that BAY reduces NLRP3’s ATPase activity and inhibits ASC oligomerization (Juliana et al., 2010). Upon further investigation, this study found that BAY inhibits NLRP3 activity by irreversibly alkylating cysteine residues in the ATPase region of NLRP3 through a Michael addition mechanism and SAR studies of BAY revealed the importance of the vinyl sulfone in the observed inhibitory activity. BAY can inhibit IL-1β production in BMDM’s with an IC_50_ of ~5 μM (Jiang H. et al., 2017). Increased ubiquitination of NLRP3 by BAY was also observed which may contribute to its inhibition of the NLRP3 protein by interfering with NLRP3-ASC interactions (Shim et al., 2017). A recent study demonstrated that BAY inhibits pore-formation of GSDMD by covalently modifying the critical cysteine residue C191 which is involved in GSDMD pore-formation (Hu et al., 2018). This study also found that BAY can inhibit canonical and non-canonical caspases. BAY has been investigated in a number of disease states and injuries including psoriasis, diabetic nephropathy, spinal cord injury, and extensively in cancer for its effects on the NF-κB pathway (White and Burchill, 2008; Kolati et al., 2015; Irrera et al., 2017; Jiang W. et al., 2017).

#### 3,4-Methylenedioxy-β-Nitrostyrene

3,4-Methylenedioxy-β-nitrostyrene (MNS) is a well-known Syk and Src tyrosine-kinase inhibitor; however, it is also shown to be a direct, selective inhibitor of the NLRP3 inflammasome (He et al., 2014). MNS can block IL-1β production with an IC_50_ of 2 μM in BMDMs. Pull-down studies suggest that MNS binds to the LRR and NACHT domains of the NLRP3 protein, and further investigation showed that the MNS inhibits NLRP3 ATPase activity. MNS has no inhibitory activity towards AIM2 or NLRC4 inflammasomes. Moreover, SAR studies of MNS indicated that the nitrovinyl group is essential for its inhibition of NLRP3, whereas the dioxole is dispensable. It’s suspected that the vinyl group may interact with the cysteine side chains in NLRP3, possibly in the ATPase region, making it an irreversible inhibitor (He et al., 2014). A recent study found that MNS induced ubiquitination of NLRP3, which may contribute to its inhibitory mechanism (Shim et al., 2017). MNS has been studied in wound healing and in osteosarcoma tumors (Messerschmitt et al., 2012; Xiao et al., 2016).

#### Tranilast

Tranilast has historically been approved to treat bronchial asthma in Japan, China, and South Korea. Tranilast was initially found to inhibit NF-κB by interfering with NF-κB and CREB-binding protein (CBP) association, but recent studies demonstrated that tranilast is a selective inhibitor that directly binds to the NLRP3 protein and inhibits IL-1β production with an IC_50_ of 25–50 μM (Spiecker et al., 2002; Huang et al., 2018). Mechanistically, tranilast binds to the NACHT domain of NLRP3, consequently inhibiting NLRP3-NLRP3 and NLRP3-ASC interactions, but not NLRP3-NEK7, inhibiting inflammasome formation and activation of caspase-1. Unlike other direct NLRP3 inhibitors, tranilast does not inhibit ATPase activity (Huang et al., 2018). Recently, tranilast has been investigated for the treatment of neuropathic pain, gouty arthritis, CAPS, and cancer, and has been suggested to be a potential therapy for vitiligo (Huang et al., 2018; Saito et al., 2018; Moore et al., 2019; Zhuang et al., 2020).

#### OLT1177 (Dapansutrile^®^)

OLT1177 is a β-sulfonyl nitrile compound that selectively blocks the NLRP3 inflammasome by inhibiting its ATPase activity, preventing NLRP3-ASC interactions and IL-1β release in J774A.1 macrophages with an IC_50_ of ~1 nM. OLT1177 has demonstrated to be a highly potent and selective inhibitor of NLRP3 since it failed to inhibit IL-1β secretion upon activation of NLRC4 or AIM2 inflammasomes (Marchetti et al., 2018a). OLT1177 has shown both *in vitro* and *in vivo* success and has recently been developed into an oral tablet and topical gel, Dapansutrile, that has proven to be safe in humans and completed phase II clinical trial for osteoarthritis of the knee by Olatec Therapeutics LLC (Marchetti et al., 2018a). Aside from clinical trials, OLT1177 has recently been tested as a potential treatment for numerous disease states including AD, autoimmune encephalomyelitis, myocardial ischemia, arthritis, and more (Marchetti et al., 2018b; Sánchez-Fernández et al., 2019; Toldo et al., 2019; Lonnemann et al., 2020).

#### CY-09

CY-09 has recently been reported to selectively and directly bind to the ATPase domain of NLRP3 and inhibit IL-1β release in BMDM cells with an IC_50_ of ~6 μM (Jiang H. et al., 2017; El-Sharkawy et al., 2020). Mutating the Walker A motif of NLRP3’s ATPase pocket abolishes the activity of CY-09, whereas mutating the Walker B motif shows no effect, suggesting the Walker A motif of the ATPase pocket as the binding site of CY-09 where it interferes with ATP binding. This is contrary to MCC950 which binds to the Walker B motif of NLRP3. ATP also competes with CY-09 to bind to NLRP3 which further confirms this interaction. Using microscale thermophoresis (MST), CY-09 binds to recombinant NLRP3 with a K_D_ of 500 nM (Jiang H. et al., 2017). CY-09 has shown to be effective in *ex vivo* and *in vivo* models for CAPS, type 2 diabetes, gout, non-alcoholic fatty liver disease (NAFLD), pain, and more (Jiang H. et al., 2017; Fan et al., 2021; Wang X. et al., 2021).

#### BOT-4-One

BOT-4-one is a benzoxathiole derivative that exhibits anti-inflammatory activities through several different mechanisms of action due to its ability to irreversibly alkylate its targets. A 2016 study found that BOT-4-one inhibits the NF-κB pathway by alkylating IKKβ, subsequently inhibiting NLRP3 expression (Lee H. G. et al., 2016). Recent studies demonstrated that BOT-4-one inhibited NLRP3 ATPase activity through alkylation (Shim et al., 2017). Furthermore, alkylation of NLRP3 by BOT-4-one can increase ubiquitination of NLRP3, which could contribute to its inhibitory mechanism. While it’s not clear whether alkylation induces ubiquitination or inhibits deubiquitination, other NLRP3 alkylators BAY and MNS also increase NLRP3 ubiquitination. BOT-4-one is able to inhibit IL-1β secretion induced by ATP with an IC_50_ of 1.28 μM and by nigericin with an IC_50_ of 0.67 μM. This compound has been investigated in arthritis, pathogenic skin inflammation, and cancer, particularly Hodgkin’s lymphoma, in animal models (Kim et al., 2011, 2016; Lee H. G. et al., 2016).

#### Methylene Blue

Methylene blue has a long history of use in medicine, from treatment for methemoglobinemia and septic shock to being used as a dye in surgeries for tissue labeling. Methylene blue is known to have anti-inflammatory, antioxidant, and neuroprotective effects through a variety of mechanisms including its non-selective inhibition of canonical and non-canonical NLRP3, NLRC4, and AIM2 inflammasomes (Ahn et al., 2017). Studies have shown that methylene blue decreases NLRP3, pro-IL-1β, and iNOS expression by interrupting transcription factors NF-κB and STAT1. Methylene blue exhibits no effects on the nuclear transportation of NF-κB and STAT1 but markedly decreases their binding to DNA, thus suggesting methylene blue’s potential interference at binding (Huang et al., 2015). The attenuation of IL-1β and caspase-1 secretion as well as ASC oligomerization when methylene blue is added after LPS suggests that methylene blue inhibits both the priming and activation step of the NLRP3 pathway. In addition, methylene blue also inhibits the activity of recombinant capase-1 (Ahn et al., 2017). Methylene blue has been investigated in inflammation in spinal cord injury, cognitive impairment from neuroinflammation, diabetic retinopathy, AD, and more (Lin et al., 2017; Hao et al., 2019; Soeda et al., 2019; Zhou et al., 2019).

#### Disulfiram (Antabuse^®^)

Disulfiram is an FDA-approved drug to treat chronic alcohol dependence through an irreversible cysteine residue modification on aldehyde dehydrogenase. However, it’s recently been discovered that disulfiram contains anti-inflammatory properties. Deng and colleagues found that disulfiram provides lysosomal protection and regulates ROS production that is independent of mitochondria, inhibiting NLRP3-dependent IL-1β secretion with an IC_50_ of ~5 μM under LPS/ATP conditions in mouse macrophages (Deng et al., 2020). In addition, disulfiram can directly inhibit canonical and non-canonical caspases (Hu et al., 2018). Recent studies demonstrated that disulfiram inhibits GSDMD pore formation by modifying Cys191 of GSDMD and, as a result, suppressing IL-1β secretion and the subsequent pyroptosis (Hu et al., 2020). Others have investigated the potential to repurpose disulfiram to treat several diseases including cancer and bacterial infections and recently has been a suggested as a potential therapy for COVID-19 infection (Viola-Rhenals et al., 2018; Frazier et al., 2019; Fillmore et al., 2021).

#### Fenamic Acids

Fenamic acid derivatives are known non-steroidal anti-inflammatory (NSAID) agents. While the primary role of NSAIDs is inhibiting prostaglandin synthesis through cyclooxygenase (COX) enzymes, recent studies have shown that fenamic acid derivatives also exhibit anti-inflammatory effects by inhibiting the NLRP3 inflammasome pathway *via* interference with the Cl^−^ volume-regulated anion channel (VRAC), a regulator of NLRP3 (Daniels et al., 2016). While flufenamic, meclofenamic, and mefenamic acids were able to inhibit IL-1β release in iBMDMs under challenging for the NLRP3 pathway, flufenamic, and mefenamic acid were unable to inhibit IL-1β under NLRC4 and AIM2 challenge, suggesting these acid derivatives are specific to the NLRP3 pathway. In addition, other COX enzyme inhibitors such as Ibuprofen and celecoxib did not affect IL-1β release (Daniels et al., 2016). These fenamic acid derivatives have been studied in a variety of disease models including AD, prostate cancer, and more (Delgado-Enciso et al., 2015; Mangan et al., 2018).

#### Fluoxetine (Prozac^®^)

Fluoxetine, also known as Prozac, is an FDA-approved drug used to treat clinical depression. Recently, fluoxetine was found to directly inhibit the NLRP3 protein, stopping inflammasome formation and subsequent IL-1β release in retinal pigmented epithelium (RPE) and macrophage cells (Ambati et al., 2021). In addition, the study demonstrated the physical interaction of fluoxetine with the NLRP3 protein by pull-down. Further binding studies found that a CY-09 probe could also pull-down NLRP3, and that excess fluoxetine was able to compete with the binding of CY-09, an NLRP3 ATPase inhibitor, suggesting these compounds share a similar mode of binding. Both CY-09 and fluoxetine contain a (trifluoromethyl)phenyl moiety and SAR studies suggest that this moiety may assist in the interaction with NLRP3’s ATPase domain. Unlike other antidepressants, fluoxetine reduced RPE degeneration *in vivo* caused by activating the NLRP3 inflammasome (Ambati et al., 2021). This may open new avenues for the use of fluoxetine; however, more research is needed to prove its potential in different disease states.

#### β-Hydroxybutyrate

β-Hydroxybutyrate (BHB) is an endogenous ligand produced in the liver and functions as an energy source during nutrient deprivation and low-carb diets. During long fasting periods or ketogenic diets, circulating concentrations of BHB can increase which is shown to be correlated with decreased immune response. Youm and colleagues showed that BHB can inhibit the NLRP3 pathway by blocking potassium efflux, an activator of NLRP3 (Youm et al., 2015). The concentrations of BHB that produced these anti-inflammatory effects *in vitro* were similar to its endogenous level after strenuous exercise or 2 days of fasting. Unfortunately, *in vivo* administration of BHB alone resulted in rapid clearance; however, when complexed with nanolipogels, BHB retained its anti-inflammatory activity, reducing NLRP3-driven neutrophil infiltration and decreasing IL-1β release at the injection site of MSU crystals (Youm et al., 2015). In LPS-primed BMDMs, BHB can inhibit NLRP3-mediated IL-1β with an IC_50_ of ~5 μM (Jiang H. et al., 2017). BHB has been studied in stress-related mood disorders, hypertension, seizures, and gout (Goldberg et al., 2017; Yamanashi et al., 2017; Chakraborty et al., 2018; Rho et al., 2019).

#### MicroRNA as Post-transcriptional Regulators of NLRP3 Expression

MicroRNA are small, non-coding single-stranded RNA molecules that regulate gene expression post-transcriptionally by RNA silencing. These molecules represent an attractive strategy for drug discovery due to their high specificity. In addition, miRNAs have multiple binding sites, also known as seeding regions, which allows them to be involved in multiple pathways. While there are several miRNAs that influence different targets in the NLRP3 inflammasome pathway, there are several miRNAs that target the NLRP3 protein (Boxberger et al., 2019). The first miRNA discovered to target NLRP3 is miRNA (miR)-223-3p which was found to negatively regulate the NLRP3 protein (Bauernfeind et al., 2012; Haneklaus et al., 2012). It’s been proposed that miR-223-3p is important for mediating protein expression in different cell types since differentiation of monocytes to macrophages resulted in a decreased expression of miR-223-3p and increased levels of NLRP3 protein. This could potentially explain why some cell lines have low responses to inflammatory NLRP3 stimuli. MiR-7-5p and miR-30e-5p were also found to negatively regulate NLRP3 protein expression *in vitro* and *in vivo* (Zhou et al., 2016; Li et al., 2018). In addition, delivery of miR-7-5p and miR-30-5p mimics were found to protect dopaminergic neurons from degeneration and decrease inflammatory cytokines through inflammasome suppression in an MPTP-induced PD mouse model. Other miRNA’s that negatively regulate NLRP3 protein expression are miR-22-3p, miR-133b-3p, miR-186-5p, and miR-495-3p which have been studied in a variety of disease states including asthma, AD, allergic inflammation, cardiac microvascular endothelial cell injury (CMEC) and more (Xiao et al., 2017; Chen M. L. et al., 2018; Zhou et al., 2018; Han et al., 2020; Guo et al., 2021). There are currently extensive efforts underway to explore the therapeutic potentials of miRNA. However, the major limitation of these molecules is their delivery and membrane permeability. Nanoparticles show promising results in delivering peptides and could provide hope for the future of miRNAs as a therapy to treat numerous diseases (Wang et al., 2015; Sezlev Bilecen et al., 2017).

#### Anakinra (Kineret^®^), Rilonacept (Arcalyst^®^), and Canakinumab (Ilaris^®^)

There are currently three FDA-approved biologics that target the NLRP3 pathway. Anakinra was the first to be developed and is a modified IL-1Ra that blocks the binding of IL-1β and IL-1α, inhibiting their signaling pathways and further inflammation. Anakinra was approved for the treatment of RA in 2001 and was later approved for the treatment of CAPS in 2013 (Calabrese, 2002; Jesus and Goldbach-Mansky, 2014). The short plasma half-life of anakinra (4–6 h) is a pitfall among patients due to required daily injections (Granowitz et al., 1992). Rilonacept is a dimeric fusion protein with decoy receptors containing extracellular residues of the two IL-1R subunits, IL-1R1 and IL-1R accessory protein (IL-1RAcP), which can bind to and neutralize IL-1β and IL-1α (Jesus and Goldbach-Mansky, 2014). With an improved half-life (7 days) compared to anakinra, rilonacept was approved for the treatment of CAPS in 2008 and was recently approved for recurrent pericarditis in 2021 (Hoffman, 2009; Fava et al., 2022). Canakinumab is an anti-IL-1β monoclonal antibody that selectively binds to and neutralizes IL-1β. With a half-life of 26 days, canakinumab is a preferred treatment option and has been approved for CAPS, juvenile idiopathic arthritis, rare periodic fever syndromes, and adult-onset Still’s disease (AOSD) (Curran, 2012; Orrock and Ilowite, 2016; Malcova et al., 2020; Sfriso et al., 2020). In addition, a number of clinical trials have recently investigated anakinra and canakinumab as a therapeutic treatment for patients with COVID-19 (Kooistra et al., 2020; Landi et al., 2020; Kyriazopoulou et al., 2021; Kharazmi et al., 2022). These therapies have proven to be safe and effective in treating inflammation-driven diseases, however, some issues remain. All three biologics require administration by injection, a route that is not preferred by patients and is particularly problematic for anakinra due to its short half-life. In addition, due to the nature of biologics and protein-based therapies, it’s likely that these therapeutics have poor BBB penetrance, potentially limiting their applications to diseases outside of the CNS. Furthermore, increased infection is a concern for therapies blocking all IL-1β signaling regardless of the source, an issue that was observed with canakinumab (Dinarello et al., 2012; Mangan et al., 2018). These problems emphasize the necessity for small-molecule inhibitors of NLRP3 to overcome pharmacokinetic and selectivity issues.

## Conclusion

The NLRP3 inflammasome plays an intricate and important role in the innate immune system in response to a variety of stimuli. Upon the formation of the NLRP3 inflammasome, caspase-1 is released and activates the pro-inflammatory cytokines IL-1β and IL-18. These cytokines continue the inflammatory signaling cascade and recruit immune cells to fight infections or heal injuries. Aberrant activation of NLRP3 leads to chronic inflammation, a common denominator of several diseases that causes and/or encourages disease pathology. NLRP3 is activated and IL-1β and IL-18 levels are upregulated in diseases where pathology is accompanied by inflammation. Thus, NLRP3 has become an attractive target in the drug discovery field and inhibitors possess high therapeutic value for the treatment of many diseases. Several hurdles remain to finding a potent and selective NLRP3 inhibitor with adequate pharmacokinetic properties, particularly BBB penetrance for CNS diseases. More research is required to further the understanding of the binding and mechanism of compounds targeting this pathway.

## Author Contributions

HB and SZ contributed to conceptualization, writing, and editing. SB and YX contributed to editing. All authors contributed to the article and approved the submitted version.

## Conflict of Interest

The authors declare that the research was conducted in the absence of any commercial or financial relationships that could be construed as a potential conflict of interest.

## Publisher’s Note

All claims expressed in this article are solely those of the authors and do not necessarily represent those of their affiliated organizations, or those of the publisher, the editors and the reviewers. Any product that may be evaluated in this article, or claim that may be made by its manufacturer, is not guaranteed or endorsed by the publisher.
